# Transcriptome Profiling of *Trypanosoma brucei* Development in the Tsetse Fly Vector *Glossina morsitans*

**DOI:** 10.1371/journal.pone.0168877

**Published:** 2016-12-21

**Authors:** Amy F. Savage, Nikolay G. Kolev, Joseph B. Franklin, Aurelien Vigneron, Serap Aksoy, Christian Tschudi

**Affiliations:** 1 Department of Epidemiology of Microbial Diseases, School of Public Health, Yale University, New Haven, Connecticut, United States of America; 2 Department of Cell Biology, School of Medicine, Yale University, New Haven, Connecticut, United States of America; Louisiana State University, UNITED STATES

## Abstract

African trypanosomes, the causative agents of sleeping sickness in humans and nagana in animals, have a complex digenetic life cycle between a mammalian host and an insect vector, the blood-feeding tsetse fly. Although the importance of the insect vector to transmit the disease was first realized over a century ago, many aspects of trypanosome development in tsetse have not progressed beyond a morphological analysis, mainly due to considerable challenges to obtain sufficient material for molecular studies. Here, we used high-throughput RNA-Sequencing (RNA-Seq) to profile *Trypanosoma brucei* transcript levels in three distinct tissues of the tsetse fly, namely the midgut, proventriculus and salivary glands. Consistent with current knowledge and providing a proof of principle, transcripts coding for procyclin isoforms and several components of the cytochrome oxidase complex were highly up-regulated in the midgut transcriptome, whereas transcripts encoding metacyclic VSGs (mVSGs) and the surface coat protein brucei alanine rich protein or BARP were extremely up-regulated in the salivary gland transcriptome. Gene ontology analysis also supported the up-regulation of biological processes such as DNA metabolism and DNA replication in the proventriculus transcriptome and major changes in signal transduction and cyclic nucleotide metabolism in the salivary gland transcriptome. Our data highlight a small repertoire of expressed mVSGs and potential signaling pathways involving receptor-type adenylate cyclases and members of a surface carboxylate transporter family, called PADs (Proteins Associated with Differentiation), to cope with the changing environment, as well as RNA-binding proteins as a possible global regulators of gene expression.

## Introduction

One of the challenges of being a successful parasitic organism cycling between different hosts is to adapt and survive in drastically different environments. For instance, the protozoan parasite *Trypanosoma brucei*, causing sleeping sickness in humans and nagana in animals [1, 2], is transmitted between different mammals by its insect vector, the blood-feeding tsetse fly (*Diptera*: *Glossinidae*). Successful completion of the life cycle, i.e. survival in and adaptation to new surroundings, requires activation of specific and complex developmental programs which culminate in the manifestation of at least 10 distinct morphological forms [3–5]. In the mammalian bloodstream, a pleomorphic strain exists as both a long slender form that can replicate by asexual division and a cell cycle arrested short stumpy form pre-programmed to encounter the insect host [6]. The transmission cycle begins with the tsetse fly taking a bloodmeal on an infected mammal. While in the midgut of the insect vector, slender forms are killed by the action of proteases and short stumpy forms differentiate into proliferating procyclics. Following establishment of midgut procyclics, trypanosomes must find their way to and colonize salivary glands [7]. This part of the life cycle in the insect vector consists of several morphological forms, including long trypomastigote forms which go through an asymmetric division producing short epimastigotes believed to colonize the salivary gland [8–10]. In this final stage of the *T*. *brucei* life cycle in the insect vector, epimastigotes are attached to the epithelium while they differentiate to pre-metacyclics and eventually arrest in G1, before being released as nascent metacyclics, again highlighting a complex differentiation process. The cycle closes when the fly takes another bloodmeal and transfers metacyclics into the bloodstream of the next mammalian host.

During the life cycle, as described briefly above, *T*. *brucei* needs to make crucial adaptations to new environments, including different temperatures and nutritional resources, and the parasites need to cope with the immune system in each host. In the mammalian bloodstream, *T*. *brucei* replicates extracellularly, and its cell surface is shielded from the environment by a densely packed coat of a single variant surface glycoprotein (VSG). Periodic switching to a different VSG enables the parasite population to evade the host immune response, thus establishing an infection which will be fatal, if not treated [11]. Upon entering the insect host, the VSG coat is replaced by procyclins, a family of glycoproteins characterized by internal Glu-Pro (EP) or Gly-Pro-Glu-Glu-Thr (GPEET) repeats, and at the same time trypanosomes lose their mammalian infectivity [12]. Next, following differentiation to epimastigotes, the surface coat changes to the brucei alanine-rich protein (BARP), so far the only specific molecular marker for proliferating parasites in the salivary glands [13]. The surface remodeling is complete by the acquisition of a VSG coat by the metacyclic forms, which re-establishes infectivity [14]. A second major change during the *T*. *brucei* life cycle occurs in the mitochondrial metabolism [15]. Parasites must successfully move from the glucose-rich bloodstream to the tsetse midgut, where proline appears to be an important energy source, although the evidence for this is vague. Thus, procyclics derive their energy mainly by metabolizing amino acids through pathways located in the mitochondrion as well as outside, whereas bloodstreams rely exclusively on glycolysis for energy production and have a mitochondrion with reduced morphological complexity.

This brief introduction only highlights a few of the adaptive changes that need to occur during the *T*. *brucei* life cycle. So far, most molecular investigations concentrated on stages that can be cultured in the laboratory, namely the bloodstream and procyclic forms. Unfortunately, this excludes several stages in the insect vector, in particular developmental forms that reacquire infectivity, and experimental work on parasites in the fly is extremely challenging, which resulted in the designation of this stage of the life cycle in a review of this topic as “the heart of darkness” [5]. However, recent technological advances provide a new avenue to interrogate trypanosomes in the insect vector. We hypothesized that differentiation and infection establishment processes in the tsetse are governed by differentially expressed parasite genes. Thus, to characterize the expression profiles of gene products during the *T*. *brucei* life cycle in the insect vector, we surveyed the transcriptome using RNA-Seq and Gene Ontology (GO) analysis.

## Materials and Methods

### Tsetse experiments

All *in vivo* experiments were performed with pleomorphic *T*. *brucei brucei* RUMP 503 and we used the *Glossina morsitans morsitans* colony maintained in the insectary at Yale University for *in vivo* experiments. All manipulations, including tsetse infections, tissue dissections and RNA isolations were done as previously described [16]. Flies were dissected after a minimum of 40 days post infection and 72 h after their last blood meal, and infected salivary gland, proventriculus and midgut tissues were collected from the same flies.

### cDNA library preparation for RNA-Seq, read processing and differential transcript expression

RNA-Seq library preparation was done following our previously published protocols [17]. RNA samples were treated with Terminator 5′-phosphate-dependent exonuclease and then with RNase-free DNase I. First-strand cDNA synthesis was done with random primers and second-strand cDNA synthesis was initiated with an oligonucleotide complementary to the spliced leader (SL) sequence present at the 5’ end of each trypanosome mRNA, but not of the fly mRNA. The use of the SL oligonucleotide for second-strand synthesis could potentially reduce the yield of double-stranded cDNA, since only a subset of cDNAs will contain the SL sequence at the 5’ end, and thus could skew the representation of reads. However, since the same library protocol was applied to all developmental samples, the results were comparable. Two, four and three independent samples, i.e. biological replicas, were prepared from midgut, proventriculus and salivary gland tissues. Libraries were sequenced on an Illumina GAII platform at the Yale Center for Genome Analysis and the reads of 75 nt in length were pre-processed using the FASTX-toolkit on the public Galaxy webserver ([18–20]; http://galaxyproject.org/) and the SL sequence was trimmed using the Lasergene 12.1 software package from DNASTAR (http://www.dnastar.com/). All processed reads were mapped to the *T*. *brucei* 11 megabase chromosomes (GeneDB version 5) using the Lasergene 12.1 software package from DNASTAR. The SeqMan NGen layout algorithm by DNASTAR relies on unique subsequences of bases, or mers, which occur in overlapping regions of fragment reads. Mers that are common to two or more fragment reads are aligned to determine the overall layout of reads. Overlapping reads have many mers in common, but only a few mers per overlapping region are needed to identify the overlap. We chose 21 nt as the minimum length of a mer (overlapping region of a fragment read), in bases, required to be considered a match when arranging reads into contigs. By default, SeqMan NGen uses a local match percentage which requires that the match percentage threshold be met in each overlapping window of 50 bases. For the alignment, a minimum aligned length 35 nt was used and a maximum of two mismatches were allowed. For alignment of reads with multiple matches in the genome, we used two different parameters, namely once, i.e. random distribution, or never, i.e. restriction to non-repeated sequences. As a normalization method, we chose RPKM (reads assigned per kilobase of target per million mapped reads), where the signal values for each experiment are divided by the total bases of target sequence divided by one thousand; and the resulting number divided by the total number of mapped reads divided by one million. RPKM calculations were restricted to open reading frames. We used the Moderated t-Test, which is similar to the Student’s t-Test, and is used to compare the means of gene expression values for two individual replicates or two groups of replicates for a given gene. Whereas the Student’s t-Test calculates variance from the data that is available for each gene, the Moderated t-Test uses information from all of the selected genes to calculate variance. In addition, we used the FDR (Benjamini Hochberg) method as the P-value adjustment method. In this method, the P-values were first sorted and ranked. The smallest value got rank 1, the second rank 2, and the largest got rank N. Then, each P-value was multiplied by N and divided by its assigned rank to give the adjusted P-values. In order to restrict the false discovery rate to 0.05, all the genes with adjusted P-values less than 0.05 were selected. The RPKM expression data were tested by Pearson correlation analysis to evaluate sampling between biological replicates, and all correlation coefficiencies between biological pairs were over 0.81, indicating good reproducibility (S1 Fig). RNA-Seq data from this study have been submitted to the NCBI Sequence Read Archive—SRA at http://www.ncbi.nlm.nih.gov/Traces/sra/sra.cgi—under accession numbers SRP002243 and SRR965341.

### Gene Ontology (GO) analysis

The functional annotation analysis of differentially transcribed genes was performed using the Gene Ontology (GO) enrichment tool on the TriTrypDB webserver (http://tritrypdb.org/). GO terms were submitted to REVIGO, a web server that summarizes and condenses long lists of GO terms by removing redundant entries [21]. The analysis was conducted on the separate lists containing the up-regulated and down-regulated transcripts.

## Results and Discussion

### The repertoire of expressed metacyclic VSG transcripts

One of the hallmarks of trypanosome developmental progression in the insect vector is the re-acquisition of infectivity, which requires the expression of metacyclic VSG (mVSG) genes. Metacyclics are heterogeneous, displaying a number of variants (metacyclic variable antigen types; MVATs), with each individual trypanosome expressing just one mVSG [22]. Estimates for the MVAT diversity, based on monoclonal antibodies, range between more than 14 and a maximum of 27 [23, 24]. However, there is also evidence that at least five MVATs are expressed at very low levels and, four of these, only sporadically [25]. What has been established is that mVSG genes occupy telomeric sites and that they are expressed from a monocistronic RNA polymerase I (Pol I) transcription unit [26, 27].

**Fig 1:**
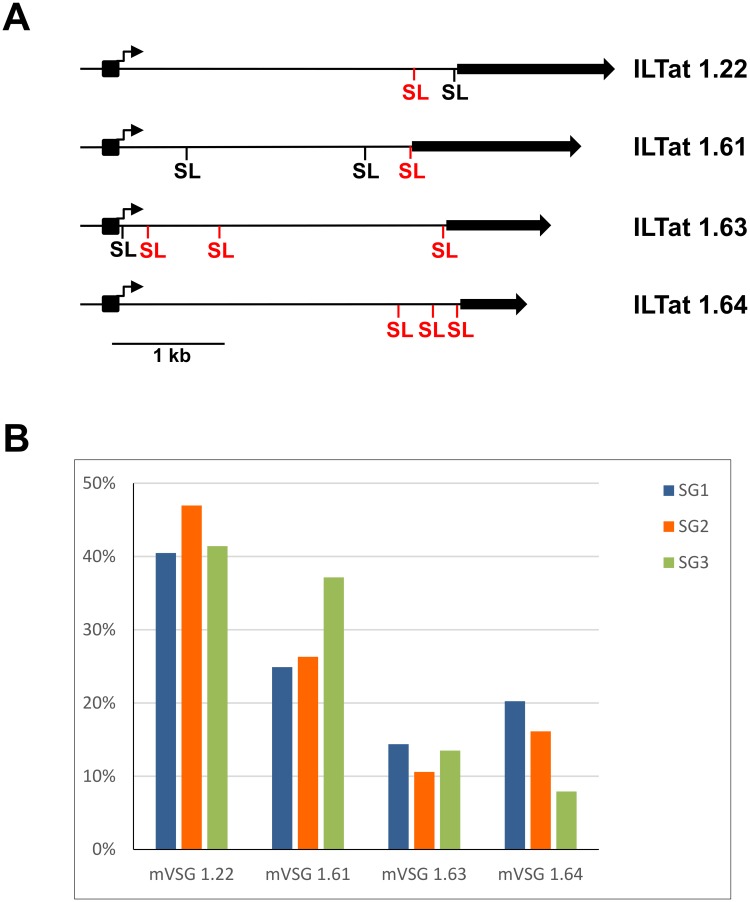
Structure and expression of the ILTat mVSG loci. (**A**) Four previously characterized mVSG loci are shown: ILTat 1.22 (ref. [28] and accession no. AJ012198); ILTat 1.61 (ref. [29] and accession no. AJ012199); ILTat 1.63 (ref. [30] and accession no. AJ486954); ILTat 1.64 (ref. [30] and accession no. AJ486955). Solid arrows, VSG genes; black boxes and arrows, mVSG promoters and transcription start sites, respectively; SL (black), position of *trans*-splices sites detected both previously by RT-PCR [30] and in the present work; SL (red), position of additional *trans*-splices sites detected in the present study. (**B**) Three independent infections with RUMP 503 were performed and the relative percentages among the four mVSGs are shown. SG1, SG2 and SG3, salivary gland transcriptomes.

To begin to address the above issue and to monitor trypanosome gene expression during differentiation in the tsetse vector on a genome-wide scale, we used a modified RNA-Seq approach (Materials and Methods and ref. [17]). Tsetse flies were infected with pleomorphic *T*. *brucei brucei* RUMP 503 parasites and following verification of the infection status by microscopic examination, trypanosomes were isolated from midgut, proventriculus and salivary gland tissues [16]. Total RNA was processed for sequencing on the Illumina platform and the resulting reads were aligned to the *T*. *brucei* 11 megabase chromosomes (release 5 of the *T*. *brucei* genome strain TREU927/4 GUTat10.1). The mVSGs expressed in *T*. *brucei brucei* RUMP 503 are not known, and the mVSG repertoire is not present in the genomic sequence of the 11 *T*. *brucei* megabase chromosomes, since they are located at telomeres. Thus, to get a handle on the mVSGs expressed in RUMP 503, reads from the salivary gland transcriptome not aligning to the 11 megabase chromosomes were assembled *de novo* and the resulting contigs were subjected to BLAST analyses of annotated *T*. *brucei* proteins at NCBI. Although the exact lineage information on RUMP 503 is not available, it appears to be a derivative of EATRO 795 fly-transmissible ILTat 1.3 (http://tryps.rockefeller.edu/trypsru2_pedigrees.html). Consistent with this history, four metacyclic ILTat VSGs were identified, namely ILTat 1.22, ILTat 1.61, ILTat 1.63 and ILTat 1.64 (Fig 1A). Re-analysis of the transcriptome data with the mVSG sequences appended revealed the four mVSG transcripts to be up-regulated between 57- to 86-fold in salivary glands compared to the proventriculus sample (S1 Table).

It is thought that each metacyclic cell randomly initiates transcription of a single mVSG gene [22]. Our RNA-Seq data revealed four prominent mVSG transcripts, although we cannot exclude the possibility of additional member(s) being expressed, since we do not have the entire repertoire available. However, what our three independent infections with RUMP 503 clearly showed was that the four mVSG transcripts emerged with comparable representations and that mVSG ILTat 1.22 was the most abundant transcript in all experiments followed by ILTat 1.61 (Fig 1B). This result is comparable to previous investigations on the composition of metacyclic populations in trypanosome stocks isolated from an East African focus of sleeping sickness, where the 1.22 mVSG (also known as GUTat 7.1) and 1.61 mVSG (GUTat 7.15) were the most commonly detected mVSGs [25].

mVSG genes are part of the only *bona fide* monocistronic transcription units for protein-coding genes and the promoter and transcription start sites for ILTat 1.22, ILTat 1.61, ILTat 1.63 and ILTat 1.64 have been mapped (Fig 1A and ref [30]). Primary transcripts are matured by coupled *trans*-splicing and polyadenylation. In *trans*-splicing, the 39-nt SL sequence is added to the 5′ end of all mRNAs and the sequence signals that determine the *trans*-splice acceptor site appear to consist only of AG dinucleotide at the site for exon junction preceded by a poly-pyrimidine tract of varying length [31]. The RNA-Seq data enabled us to map precisely the 5’ boundaries of the mVSG mRNAs by inspecting the aligned data for regions of rapid changes in the abundance of RNA-Seq tags with the concomitant identification of an AG dinucleotide of the *trans*-splice acceptor site. In all four mVSGs the most prominent 5’ boundary was very close to the AUG initiation codon (Fig 1A). Nevertheless, we also mapped SL addition sites immediately downstream of the promoter in three cases and these transcripts have been detected previously by RT-PCR in RNA isolated from tsetse-derived metacyclic trypanosomes [30]. Our read coverage was high enough to determine that these transcripts extended from the promoter all the way to the coding region, but it should be noted that the abundance of these transcripts is significantly lower when compared to those covering the ORF (S2 Fig). At present the significance of the stable transcripts downstream of the promoter in mVSG expression remains to be determined.

### Estimation of transcript abundance in trypanosomes colonizing different tsetse tissues

Biological replicates of trypanosomes isolated from the midgut, proventriculus and salivary gland were analysed by RNA-Seq for their global expression profile (see Materials and Methods). 10,239 *T*. *brucei* annotated and predicted genes were surveyed and transcripts were detected in at least one dataset for 9,699 genes, with 8,759 of these transcripts (90%) having at least 5 RPKM. The vast majority of genes with missing values corresponded to VSG pseudogenes and unlikely hypothetical proteins. The abundance of each transcript is summarized in S2 Table. In general, we achieved very good coverage of the overall transcriptome, i.e. we detected uniform coverage along the 11 mega-chromosomes and the majority of transcription units, with the exception of the subtelomeric regions. The procyclin isoforms (EP1, EP2 and EP3) were by far the most abundant transcripts in the midgut dataset (Table 1 and S3 Table).

**Table 1:** Most abundant transcripts in midgut, proventriculcus and salivary gland data sets.

Midgut		Proventriculus		Salivary Gland	
Product	RPKM	Product	RPKM	Product	RPKM
EP3-2 procyclin (EP3-2)	49,682	EP3-2 procyclin (EP3-2)	6,162	hypothetical protein	7,679
EP1 procyclin (EP1)	27,453	arginine kinase (AK)	3,860	hypothetical protein	6,802
EP2 procyclin (EP2)	20,534	beta tubulin	3,017	ILTat_1.22_mVSG	5,216
60S ribosomal protein L38	10,013	unspecified product	2,800	ILTat_1.64_mVSG	4,488
60S ribosomal proteins L38	7,599	calpain-like protein	2,758	beta tubulin	4,235
beta tubulin	6,115	60S ribosomal protein L4	2,660	inhibitor of cysteine peptidase	4,215
unspecified product	5,789	histone H3, putative	2,614	hypothetical protein, conserved	4,037
60S ribosomal protein L28	5,653	hypothetical protein	2,485	BARP protein (BARP)	3,952
60S ribosomal protein L30	5,285	hypothetical protein	2,472	ILTat_1.61_mVSG	3,261
60S ribosomal protein L39	5,248	EP2 procyclin (EP2)	2,311	60S ribosomal protein L4	3,254
pteridine transporter	5,157	histone H4, putative	2,208	60S ribosomal proteins L38	3,100
60S ribosomal protein L44	4,790	EP1 procyclin (EP1)	2,192	40S ribosomal protein S5	3,088
60S ribosomal protein L22	4,667	alpha tubulin	1,889	60S ribosomal protein L38	3,044
ribosomal protein S26	4,584	beta tubulin, pseudogene	1,825	60S ribosomal protein L44	2,974
40S ribosomal protein S5	4,471	60S ribosomal protein L22	1,775	alpha tubulin	2,959
hypothetical protein	4,265	pteridine transporter	1,729	beta tubulin, pseudogene	2,917
60S ribosomal protein L35	4,237	40S ribosomal protein S5	1,721	ILTat_1.63_mVSG	2,496
60S ribosomal protein L4	4,216	60S ribosomal proteins L38	1,623	60S ribosomal protein L27a	2,330
60S ribosomal protein L27a	4,209	60S ribosomal protein L39	1,582	amino acid transporter 1 (AATP1)	2,275
60S ribosomal protein L35	4,200	histone H1	1,561	40S ribosomal protein S15	2,238
60S ribosomal protein L27	4,146	60S ribosomal protein L38	1,512	60S ribosomal protein L28	2,234
amino acid transporter	4,114	60S ribosomal protein L28	1,445	60S ribosomal protein L27	2,207
60S ribosomal protein L32	3,982	ribosomal protein S26	1,388	Nascent polypeptide subunit beta	2,201
60S ribosomal protein L35a	3,878	60S ribosomal protein L44	1,354	60S ribosomal protein L29	2,193
40S ribosomal protein S17	3,760	60S ribosomal protein L32	1,319	60S ribosomal protein L24	2,157
40S ribosomal protein S12	3,574	60S ribosomal protein L6	1,271	60S ribosomal protein L22	2,142
40S ribosomal protein S11	3,478	translation initiation factor 5A	1,262	ribosomal protein L36	2,106
conserved protein	3,296	heat shock protein 70	1,262	60S ribosomal protein L35a	2,047
ribosomal protein S19	3,177	60S ribosomal protein L31	1,246	PAD4	2,031
60S acidic ribosomal protein	3,158	60S ribosomal protein L9	1,210	ribosomal protein S7	1,993
40S ribosomal protein S15	3,068	amino acid transporter (AATP11)	1,203	histone H1	1,962
beta tubulin, pseudogene	3,056	60S ribosomal protein L13a	1,169	ubiquitin/ribosomal protein S27	1,955
40S ribosomal protein SA	3,042	40S ribosomal protein S12	1,154	60S acidic ribosomal protein	1,955
40S ribosomal protein SA	3,029	ribosomal protein S19e	1,148	40S ribosomal protein S16	1,953
hypothetical protein	3,019	cytochrome oxidase subunit VI	1,137	60S ribosomal protein L30	1,918
hypothetical protein	2,976	ZC3H36	1,132	histone H3	1,913
ribosomal protein S7	2,961	60S ribosomal protein L35	1,132	60S acidic ribosomal protein P2	1,908
40S ribosomal proteins S11	2,913	hypothetical protein	1,117	60S ribosomal protein L34	1,907
NRBD2	2,899	ribosomal protein S7	1,116	amino acid transporter	1,903

See S3 Table for complete list of top 200 and accession numbers.

Nevertheless, among the 200 most highly expressed mRNAs in the midgut transcriptome, the vast majority were coding for ribosomal proteins (115). Other highly expressed mRNAs originated from genes coding for six amino acid and nucleoside transporters, α- and β-tubulin, four cytochrome oxidase subunits, two RNA binding proteins essential for ribosomal assembly (NRBD1, Tb0927.11.14000, and NRBD2, Tb927.11.14020, aka p34 and p37, ref. [32]) and 27 hypothetical proteins of unknown function. It is worth noting that two transcripts encoding isoforms 1 (Tb927.7.5930) and 2 (Tb927.7.5940) of proteins associated with differentiation or PADs (see below) were in the top 200. Transcripts coding for ribosomal proteins (114/200) and procyclins remained dominant in the proventriculus sample, but one noticeable change was the emergence of transcripts encoding histones H1 to H4 (Tb927.11.1880, Tb927.7.2870, Tb927.1.2550 and Tb927.5.4180), and a transcript encoding a putative zinc finger protein ZC3H36 (Tb927.10.12760). In the salivary gland sample procyclin transcripts were replaced by the brucei alanine-rich protein called BARP [13] and metacyclic VSG as highly expressed transcripts encoding surface proteins. It should be noted that transcripts encoding BARP, a molecular marker for proliferating epimastigotes [13], were as abundant as mVSG transcripts, indicating that the salivary gland transcriptome most likely represented a mixture of epimastigotes and metacyclics. Transporters (5), histones (4), and α- and β-tubulin remained highly abundant, whereas cytochrome oxidase subunits diminished. Finally, PAD1 and PAD2, present in the top 200 in the midgut sample were diminished, but three transcripts encoding isoforms 4 (Tb927.7.5960), 6 (Tb927.7.5980) and 8 (Tb927.7.6000) of proteins associated with differentiation appeared in the list of highly expressed transcripts in the salivary gland data set.

### Summary of differential transcript expression and Gene Ontology (GO) analysis

**Fig 2:**
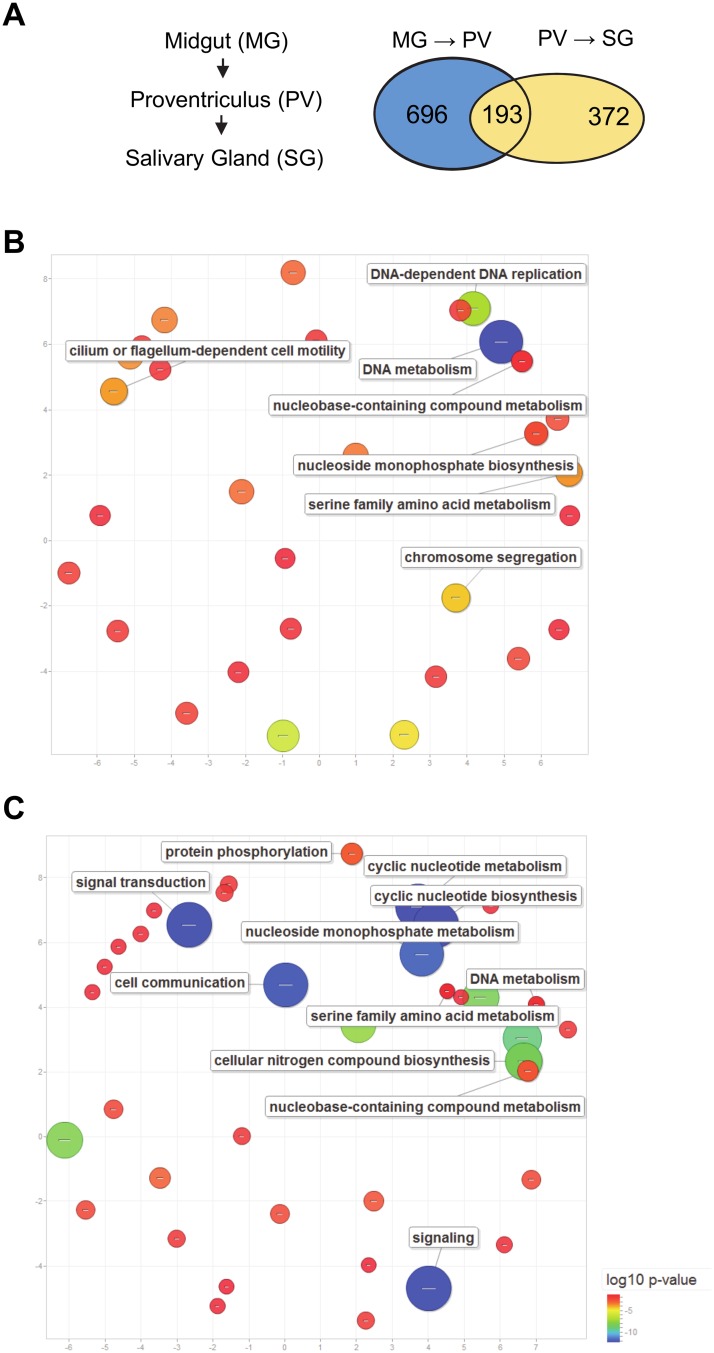
GO enrichment of differentially expressed genes between developmental stages. (**A**) A venn diagram of the number of unique and common differentially expressed transcripts between stages. (**B**) Scatterplot of enriched GO terms in up-regulated transcripts in the proventriculus. (**C**) Scatterplot of enriched GO terms in down-regulated transcripts in the salivary gland.

To begin to probe potential molecular differences between trypanosomes isolated from the three developmental stages, we defined differential expression as significant when transcript abundance changed at least two-fold and with a q-value <0.05 (FDR Bonferroni corrected) between two different stages. With this criteria and following filtering of pseudogenes, retrotransposon hot spot (RHS) protein genes and unlikely hypothetical proteins, 1,454 (15%) transcripts revealed significant differential expression in at least one developmental stage comparison (Table 2). In the proventriculus sample 889 transcripts changed abundance relative to the midgut sample, with 670 and 219 transcripts up- and down-regulated, respectively (S1 Table). Although 295 (44%) of the up-regulated transcripts encoded hypothetical proteins, using functional categorization by Gene Ontology (GO) analysis we were able to identify significantly enriched GO terms. Notably, categories involved DNA metabolism, DNA replication, transport and cillary and flagellar motility were at the top of the list (Fig 2B; see S4 Table for full list of GO terms). This is likely a reflection of proliferating cells, which is also evident in the proventriculus set of most abundant transcripts with the appearance of several histone transcripts. GO analysis of the differentially down-regulated transcripts revealed statistically significant biological processes, including carbohydrate catabolism and several transport processes (S4 Table).

**Table 2:** Differentially expressed transcripts.

	Proventriculus/Midgut	Salivary gland/ Proventriculus
**Significantly regulated transcripts**	889	565
**Up-regulated**^1^	670	238
**Down-regulated**^2^	219	327

^1^up-regulated in the proventriculus when compared to the midgut and salivary gland vs. proventriculus

^2^down-regulated in the proventriculus when compared to the midgut and salivary gland vs. proventriculus

In the salivary transcriptome 565 transcripts revealed a significant change, when compared to the proventriculus sample, with 238 transcripts up-regulated, whereas 327 transcripts were down-regulated (Table 2 and S1 Table). GO analysis revealed statistically significant up-regulation of biological processes, including plasma membrane organization, carbohydrate catabolism and several different transport processes. In contrast, in the down-regulated set of transcripts there was very a significant enrichment of categories like signal transduction, cell communication, cyclic nucleotide metabolism and DNA metabolism (Fig 2C).

### Differential expression of transcripts encoding surface proteins

**Fig 3:**
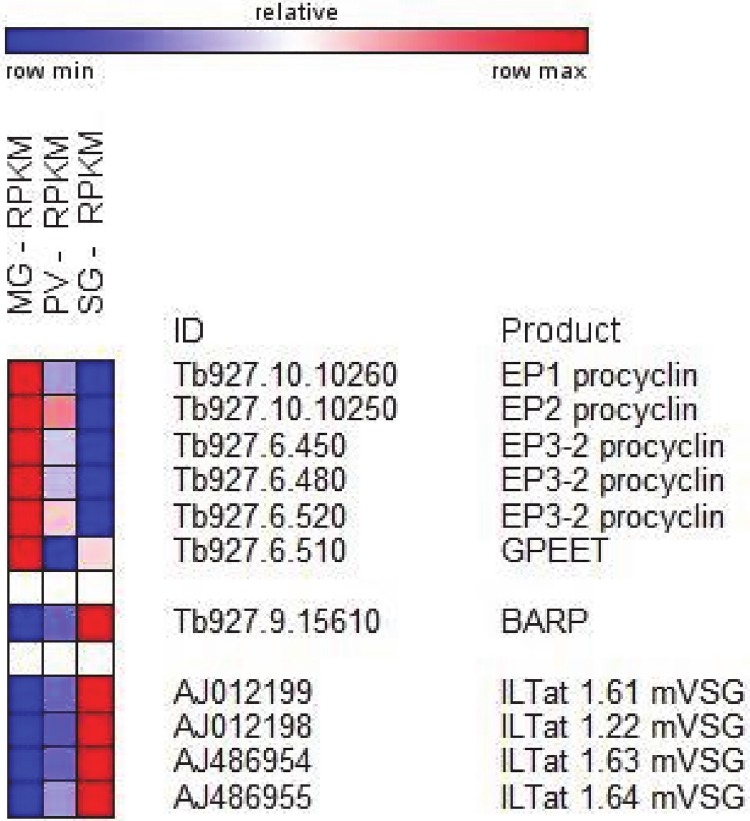
Major changes in transcript abundance during *T*. *brucei* development in the insect vector of selected surface proteins. Heatmap showing RPKM changes between midgut (MG), proventriculus (PV) and salivary gland (SG). Heatmap generated with GENE-E (http://www.broadinstitute.org).

The most striking and best understood changes occurring during the trypanosome developmental progression in the insect vector are at the level of the parasite surface. The procyclic form, residing in the midgut is covered with a procyclin coat, whereas epimastigote forms mainly have a coat consisting of BARP [13]. Finally, the infectious metacyclics living in the salivary gland express a single mVSG. Paralleling this protein knowledge, transcripts coding for EP1, EP2 and EP3 procyclin isoforms [33] were highly up-regulated in the midgut transcriptome compared to the other two stages (Fig 3). The abundance of these transcripts decreased steadily during developmental progression with an average 12-fold down-regulation in proventriculus as compared to midgut trypanosomes. On the other hand, transcripts for the GPEET isoform, which coats early procyclic forms [33], were much less abundant and although this value decreased progressively, it was less than two-fold between the three transcriptomes. Transcripts encoding BARP, a specific marker for proliferating epimastigotes [13], were 87-fold up-regulated in the salivary gland transcriptome compared to the midgut sample (Fig 3 and S1 Table). In contrast, these transcripts were only slightly up-regulated in the proventriculus transcriptome (5.6-fold), as compared to the midgut. As described above, the four identified mVSG transcripts were up-regulated between 57- to 86-fold in salivary glands compared to the proventriculus sample. Thus, generally the transcriptome data for the major known surface coat proteins closely mirrored protein expression.

Overall, there was a clear trend for differentially expressed transcripts to encode proteins of the African trypanosome cell surface phylome or CSP [34]. Out of 1,454 transcripts 7.8% (192) encode proteins of the CSP. In particular, among the 238 transcripts up-regulated in the salivary gland transcriptome when compared to the proventriculus sample, 71 (30%) transcripts encode proteins of the CSP (S1 Table). In addition to BARP and mVSGs described above, included were transcripts encoding 12 invariant surface glycoproteins, 2 atypical VSGs, 7 transporters, including the bloodstream-specific glucose transporter 1B [35], several expression-site associated genes (ESAGs) and PAD4, 6 and 8 (see below). The invariant surface glycoproteins have been described more than 20 years ago [36] and although it was recently shown that a bloodstream stage-specific ISG75 family mediates suramin uptake [37], these invariant proteins remain poorly characterized and it is not known what role, if any, they play during the trypanosome development cycle. Atypical VSGs are another family of poorly characterized surface proteins and they were named atypical, because they lack cysteine residues in the C terminus [38]. Finally, the salivary gland CSP data set revealed four up-regulated transcripts encoding hypothetical proteins with predicted GPI anchors (Tb927.7.360, Tb927.7.380, Tb927.7.400, Tb927.7.420), that we previously showed by RT-PCR to be up-regulated in trypanosomes residing in salivary glands [16, 39].

One of the best studied differentiations in *T*. *brucei* is the transition from stumpy forms to procyclic trypomastigotes, which involves uptake of citrate/*cis*-aconitate (CCA) by surface-associated carboxylate-transporter family members, namely Proteins Associated with Differentiation or PADs [40]. PAD1 is only expressed at significant levels in stumpy forms and PAD2 is thermoregulated. CCA also appears to affect the activity of *Tb*PTP1, a phosphotyrosine phosphatase, which prevents the development from stumpy to procyclic forms [41]. A downstream substrate for *Tb*PTP1 is a glycosomal directed serine/threonine-specific phosphatase, *Tb*PIP39, which promotes differentiation when it becomes phosphorylated as a consequence of the inhibition of *Tb*PTP1 [42]. Intriguingly, whereas PAD1, PAD2, PAD3, PAD5 and PAD7 were down-regulated between 3.9- and 6.2-fold in the proventriculus transcriptome when compared to that of the midgut, PAD4, PAD6 and PAD8 were 5.4-, 6.0- and 4.5-fold up-regulated, respectively, in trypanosomes residing in salivary glands when compared to the proventriculus transcriptome (S1 Table).

### Differential expression of transcripts encoding receptor-type adenylate cyclases (ACs)

**Fig 4:**
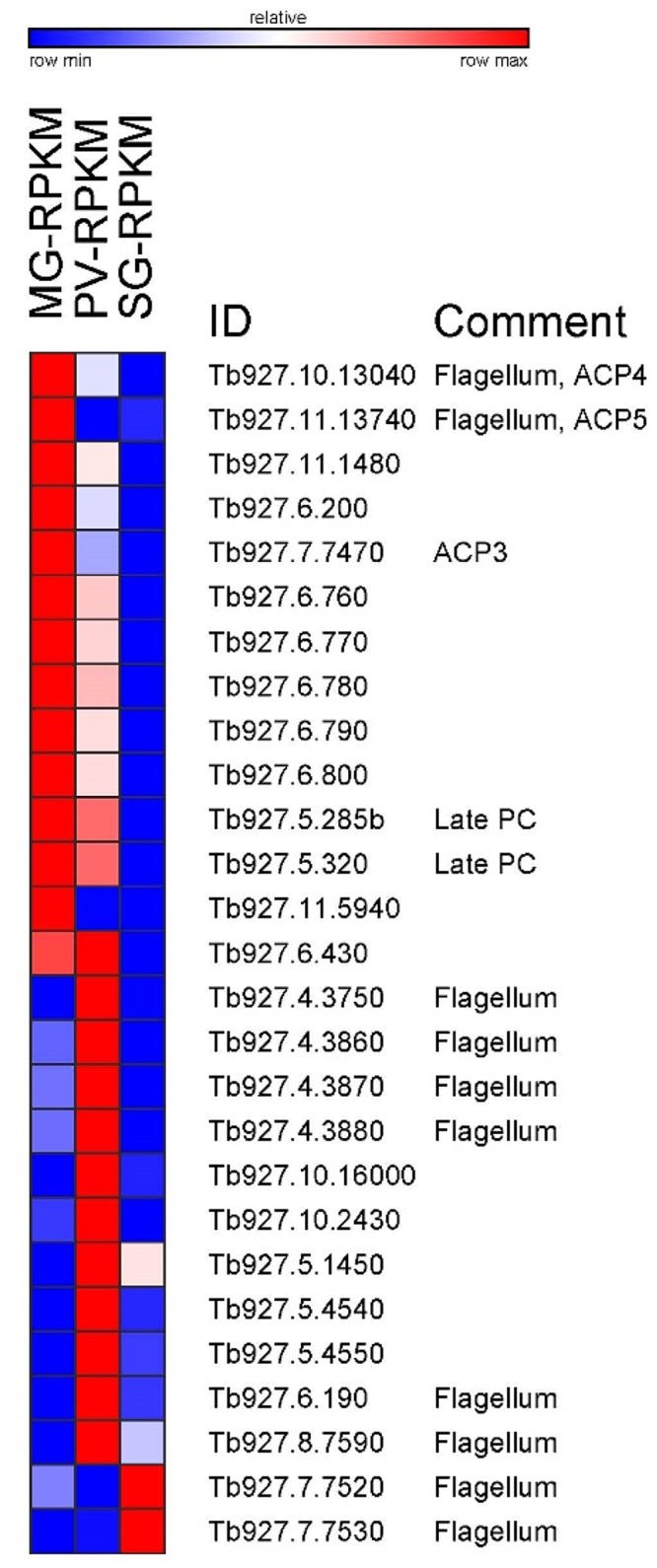
Changes in transcript abundance during *T*. *brucei* development in the insect vector of receptor-type adenylate cyclases. Heatmap showing RPKM changes between midgut (MG), proventriculus (PV) and salivary gland (SG). Heatmap generated with GENE-E (http://www.broadinstitute.org). Comments based on references [47, 50, 52].

The extracellular life cycle of *T*. *brucei* requires a mechanism to sense the environment and respond to it appropriately. It is likely that this involves a repertoire of receptors and signaling pathways and there is some evidence that cAMP may play an important role in this cascade. What has been documented is an unusual expansion of transmembrane receptor-like adenylate cyclases (ACs) with the *T*. *brucei* genome encoding more than 80 ACs [43]. This expansion is only seen in extracellular African trypanosomes, but not in related kinetoplastids, like *T*. *cruzi* and *Leishmania*, which live intracellularly. One well-studied subfamily of ACs is encoded by one of the expression site associated genes, namely ESAG4, is bloodstream specific, localizes to the flagellar membrane and is involved in host-parasite interaction [44–47]. Most of these ACs are referred to as genes related to ESAG4 (GRESAG4) and previous studies have revealed a spectrum of expression patterns ranging from constitutive transcription throughout the life cycle of the parasite to specific up-regulation in either bloodstream or procyclic trypanosomes [48, 49]. Inspection of the transcriptomes revealed three distinct sets of transcripts encoding ACs. The first group of 14 ACs was highly up-regulated in trypanosomes isolated from the midgut (Fig 4) and their abundance decreased steadily in the proventriculus and salivary gland transcriptome. Among the 14 ACs we identified as being up-regulated in procyclics are three (Tb927.7.7470, Tb927.10.13040, and Tb927.11.13740), which have been shown previously to be specifically expressed in this stage of the life cycle and they were also shown to be localized to the flagellar membrane [50]. Two additional midgut-specific ACs (Tb927.5.285b and Tb927.5.320) have been identified as markers for late procyclic forms [51]. A second and different set of 13 ACs was most prominently expressed in the proventriculus transcriptome with several members previously appearing in flagellum-specific proteomes (Fig 4). Lastly, two ACs (Tb927.7.7520 and Tb927.7.7530) were specifically overexpressed in the salivary gland transcriptome and previous evidence has shown that they are localized to the flagellum [47]. Taken together, our results provide evidence for the importance of distinct ACs when trypanosomes encounter different environments in the tsetse fly.

### Additional transcript abundance variations highlight developmental changes

Drastically different nutrient availability in the mammalian and insect host necessitates trypanosomes to adjust its energy metabolism [53]. In the midgut of the insect vector trypanosomes rely on oxidative phosphorylation, this occurring within a highly-branched mitochondrion, whereas a switch to glycolysis for ATP production takes place in bloodstream-form trypanosomes, responding to an environment rich in glucose. This adaptation is accompanied by a reduction in size and complexity of the mitochondrion and the expression of high-capacity glucose transporters on the surface of the parasite. The cytochrome oxidase (COX) complex, comprising > 10 subunits, is a key control point in respiratory activity [53, 54]. In contrast, bloodstream-form cells appear not to use a cytochrome-mediated respiratory chain and instead use an alternative pathway consisting of glycerol-3-phosphate dehydrogenase and trypanosome alternative oxidase (TAO). Among the 327 transcripts significantly down-regulated in the salivary gland transcriptome were 22 transcripts encoding known or predicted mitochondrial proteins (S1 Table), including cytochrome oxidase subunit IV (COXIV, Tb927.1.4100), cytochrome oxidase subunit VII (COXVII, Tb927.3.1410), cytochrome oxidase subunit V (COXV, Tb927.9.3170) and cytochrome oxidase subunit IX (COXIX, Tb927.10.8320). On the other hand, the transcript encoding glycerol-3-phosphate dehydrogenase (Tb927.11.7380) increased 2.9-fold from midgut to the proventriculus and then slightly decreased in the salivary gland transcriptome and TAO (Tb927.10.7090) was up-regulated 22-fold in salivary glands as compared to the proventriculus. Taken together, and considering that the salivary transcriptome represents a complex mixture of epimastigotes and metacyclics, our data nevertheless go along with observed morphological changes in mitochondrial structure and upcoming adaptations to the bloodstream environment.

**Fig 5:**
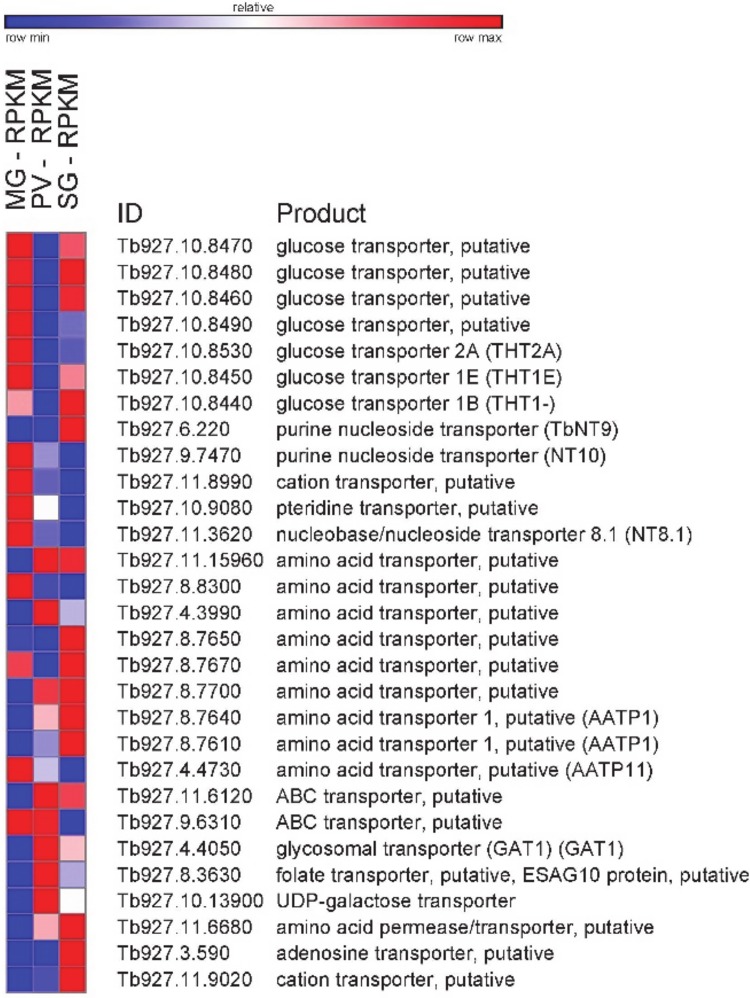
Changes in transcript abundance during *T*. *brucei* development in the insect vector of transporters. Heatmap showing RPKM changes between midgut (MG), proventriculus (PV) and salivary gland (SG). Heatmap generated with GENE-E (http://www.broadinstitute.org).

In addition to changes in energy metabolism, different nutrient availability in the insect host could lead trypanosomes to modulate expression levels of various transporters. Indeed, transcripts encoding known and predicted transporters varied significantly in the three transcriptomes (Fig 5). Notably, most glucose transporter transcripts, including the bloodstream form—specific transporter 1B (Tb927.10.8440), were initially high, decreased in the proventriculus transcriptome and then resurfaced in the salivary gland transcriptome.

### Comparison of the transcriptome from midgut-derived and culture adapted procyclic-form trypanosomes

With the caveat that we do not have a procyclic strain derived from RUMP 503, the strain we used for our fly infection experiments, we compared our previous RNA-Seq data from procyclic form *T*. *brucei rhodesiense* YTat 1.1 [17] to the transcriptome of trypanosomes isolated from the midgut (S5 Table). 82 transcripts were up-regulated in the midgut sample, including the procyclin isoforms (EP1, EP2 and EP3), PAD2, 6 and 8, and several receptor-type adenylate cyclases. 73 transcripts were down-regulated in the midgut sample, among them were transcripts encoding the GPEET isoform, histones H1, H2A and H4, and several components of the flagellum. It is worth noting that there was a scarcity of RBPs, with only two putative zinc finger proteins present (Tb927.10.12760 and Tb927.7.690).

### How is transcript abundance regulated between life-cycle stages?

**Fig 6:**
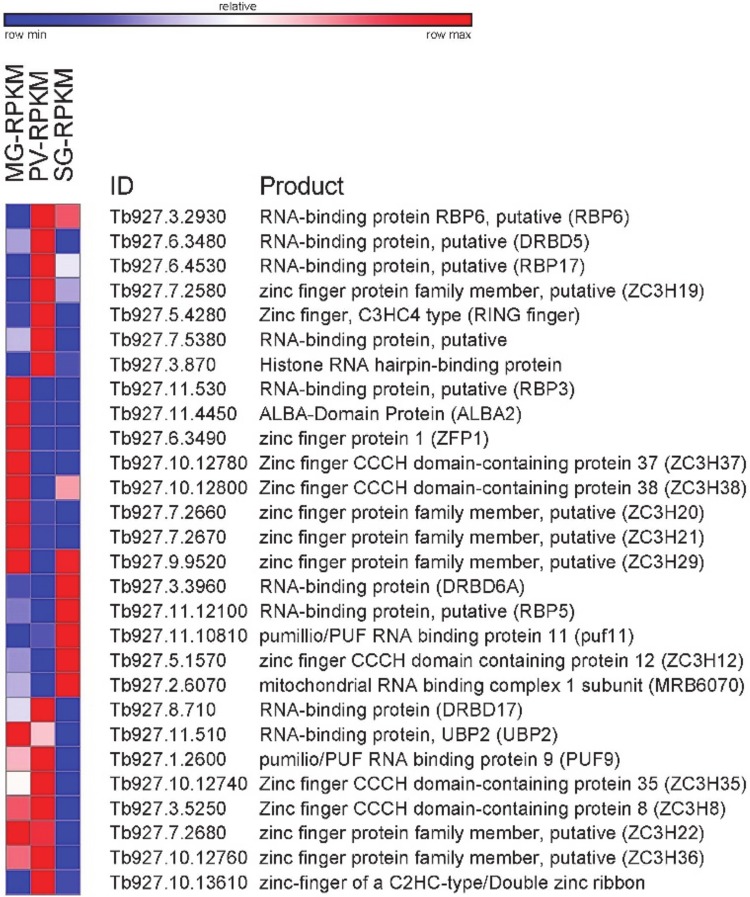
Changes in transcript abundance during *T*. *brucei* development in the insect vector of RNA-binding proteins (RBPs). Heatmap showing RPKM changes between midgut (MG), proventriculus (PV) and salivary gland (SG). Heatmap generated with GENE-E (http://www.broadinstitute.org).

The presented catalogue of differentially expressed transcripts did not expose genes involved in the expression of protein-coding genes, i.e. components of the transcriptional apparatus. This goes along with the generally accepted view that *T*. *brucei* and related kinetoplastid organisms do not appear to employ transcriptional control to regulate the abundance level of mRNAs and adaptation to different environments. Although various post-transcriptional mechanisms come to mind, in *T*. *brucei* there is emerging evidence that RNA-binding proteins (RBPs) play a central role in the regulation of gene expression [55]. A survey of the RNA-Seq data revealed 28 RBPs that were significantly up- or down-regulated in one of the three data sets (Fig 6). Most of these RBPs are still awaiting functional assignment, but notably the list includes ZFP1 (Tb927.6.3490) and ZC3H20 (Tb927.7.2660), two CCCH-family proteins, and ALBA2 (Tb927.11.4450), which were expressed most abundantly in the midgut transcriptome. ZFP1 was one of the first trypanosome RBPs to be implicated in development [56], was shown to be transiently upregulated during the differentiation from bloodstream to procyclic forms and consistent with our data was expressed at higher levels in established procyclic cells. ALBA2 was previously identified to interact with regulatory elements in the 3’ UTR of GPEET procyclin mRNA [57] and ZC3H20 is required for growth of procyclic forms [58], which fits our transcriptome data. Another noteworthy RBP is PUF9 (Tb927.1.2600), which was most prominent in the proventriculus transcriptome. This stage of development was significantly enriched for GO terms involved in DNA metabolism and DNA replication (Fig 2), which fits with PUF9’s known function to bind and stabilize a small number of mRNAs that increase in the late G1 phase of the cell cycle [59].

To begin to address the potential function of the identified RBPs in trypanosome development in the insect vector, we chose RBP6 (Tb927.3.2930), whose transcript was up-regulated in the proventriculus compared to the midgut and the transcript level remained elevated in the salivary gland (Fig 6 and S1 Table). RBP6 is a 239 amino acid-long RNA Recognition Motif or RRM protein. Using the pLEW100 vector, we previously overexpressed RBP6 in cultured non-infectious procyclics (Lister 427 29.13.6 cells expressing the TET repressor and T7 RNA polymerase). We showed that doxycycline induction of RBP6 over the course of 9–10 days resulted in the appearance of developmental stages that have been previously described in tsetse [60]. This serendipitous discovery not only strengthens the mechanistic role RBPs play in the regulation of trypanosome gene expression, but also provides a unique system to analyze at the molecular level the events triggered by RBP6 leading to epimastigotes and then to infectious metacyclics.

## Conclusions

The developmental program occurring in the insect vector ensures that trypanosomes advance in their life cycle and at the same time become a successful pathogen, i.e. they are adapted to the specific nutritional environment, as well as to develop into the infectious metacyclic form. We have provided a global transcriptome study of *T*. *brucei* during its travel through the insect vector by monitoring transcript abundance in trypanosomes isolated from three distinct tissues, i.e. the midgut, proventriculus and the salivary gland. In addition, this approach has very recently been extended to highlight the host-parasite interactions in the tsetse salivary glands [39]. Although we recognize that the salivary gland trypanosome transcriptome is rather complex and impossible to tease apart, since it contains data from proliferating epimastigotes as well as cell-cycle arrested and transmission competent metacyclics, the presented RNA-Seq datasets constitute the first resource of its kind that will facilitate further interrogation of “heart of darkness” *T*. *brucei* life-cycle stages in the tsetse vector.

## Supporting Information

S1 Fig Correlation coefficients. (TIF)

S2 Fig ILTat 1.64 mVSG Locus. (TIF)

S1 Table Differently Expressed Transcripts 2-fold or Higher and p<0.05. (XLSX)

S2 Table All Mapped Transcripts with Biological Replicates. (XLSX)

S3 Table Top 200 most Abundant Transcripts in Midgut, Proventriculus and Salivary glands. (XLSX)

S4 Table GO Analysis. (XLSX)

S5 Table Comparison of the transcriptome from midgut-derived and culture adapted procyclic-form trypanosomes. (XLSX)

S6 Table Analysis of the data sets published previously by Telleria et al. 2014 [39]. (XLSX)
