# Supplementary figures and images for: Transcriptome Profiling of Trypanosoma brucei Development in the Tsetse Fly Vector Glossina morsitans
Source: PLoS One. 2016 Dec 21;11(12):e0168877. doi: 10.1371/journal.pone.0168877 (PMC5176191; doi:10.1371/journal.pone.0168877)

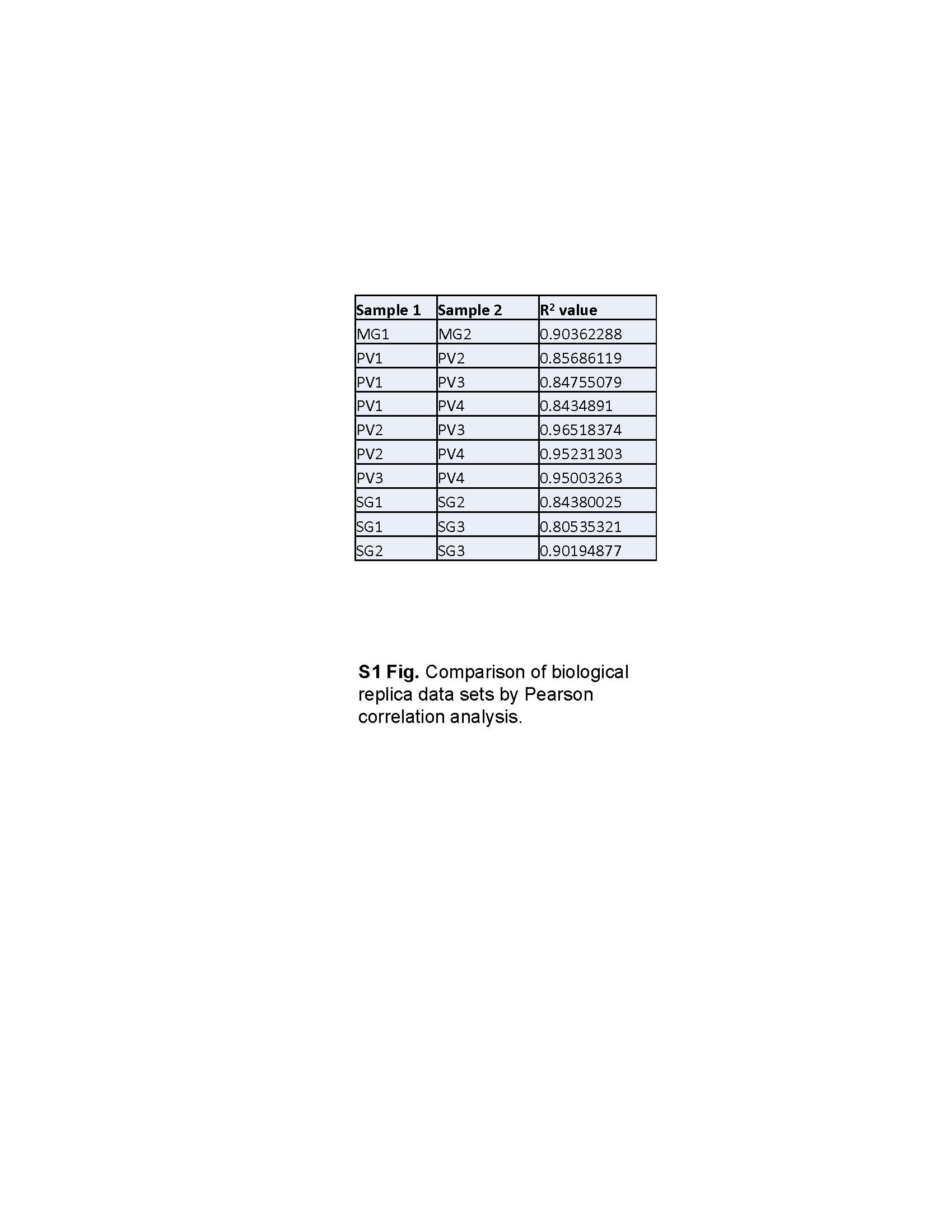

Supplement: S1 Fig — (TIF) [file pone.0168877.s001.tif]

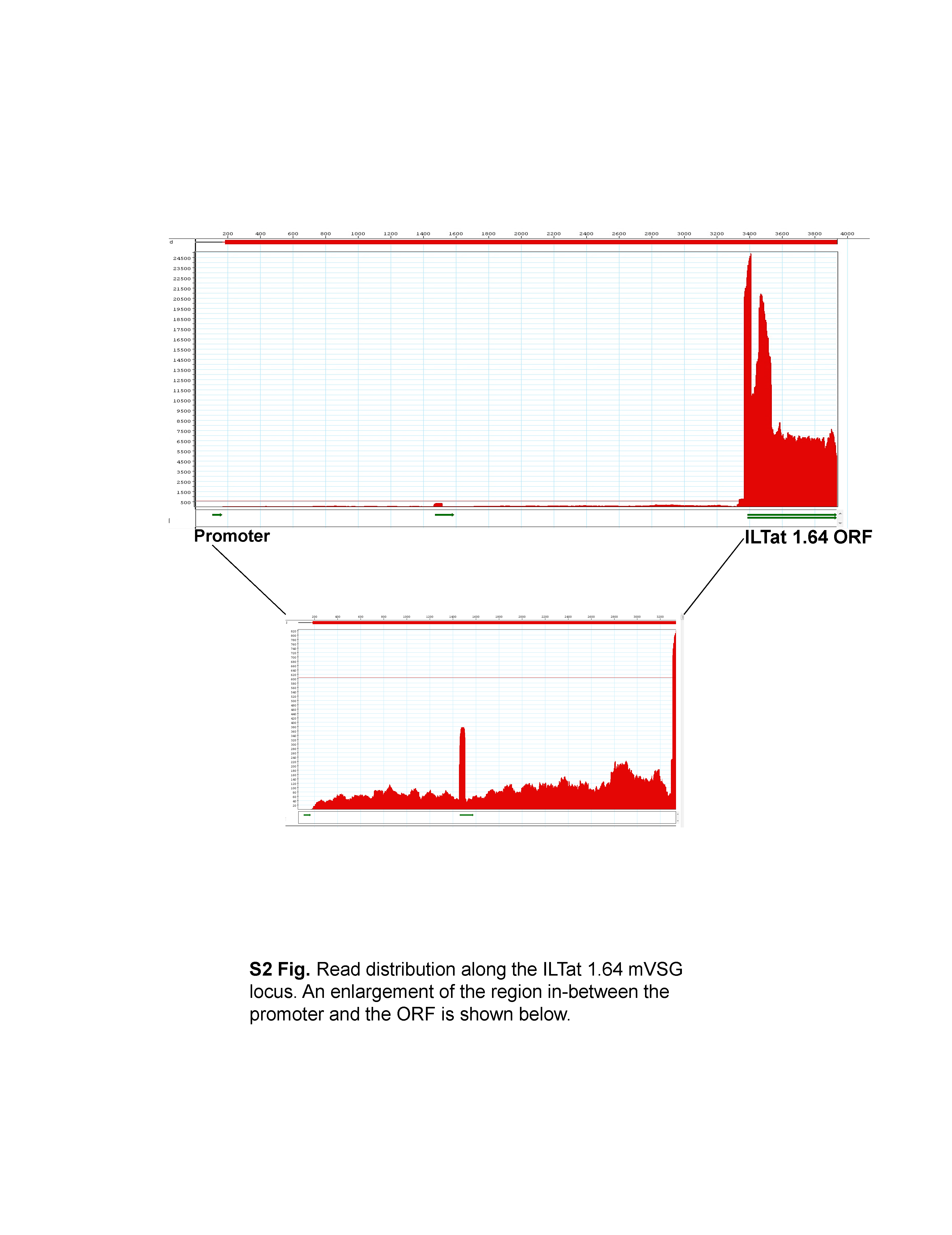

Supplement: S2 Fig — (TIF) [file pone.0168877.s002.tif]
